# Topics of Public Interest

**Published:** 1912-03

**Authors:** 


### TOPICS OF PUBLIC INTEREST

---

### The Tuberculosis Department of the Erie County Hospital

AFTER careful consideration by a special committee of the staff, this department was organized at a meeting of the staff, Feb. 5, 1912. For the present, there will be a head nurse and two assistants, with temporary assignments of pupil nurses from the main hospital; two internes, each acting as assistant and as chief, for three months each service; six attending physicians, two on duty at one time, for services of four months, each being required, personally or by assistant, to make visits at least three times a week; the whole being under one executive physician who shall visit the hospital at least twice a week. Attendants may or may not be members of the general staff but shall not serve on the general staff at the same time as in this department. Various details of management are provided for and a general provision for co-peration with the officials of the main hospital is made.

---

### Public Health and Marine Hospital Service

An examination of candidates for the position of assistant surgeon will be held in Washington, April 8, 1912. Details will be furnished on application to the Surgeon-General. This is, in many respects, the most desirable of the government services, for three reasons: officers are on duty mainly in large cities; they deal with actual patients instead of soldiers or sailors furnishing little clinical material except in venereal practice; except at the beginning, officers are not under direct supervision of superiors and, throughout, their superiors are professional colleagues. Counting quarters or allowances for quarters where none are provided, the pay amounts to about $2,000 a year at the beginning and is advanced progressively to about $4,000 in twenty years, with a chance for further promotion for a smaller number, of especial fitness.

---

"The Monroe county laboratory was established, practically doing its first year's work in 1910, and has now completed its second year of 1911, with the very creditable record that is shown in the table. In the case of Monroe county, no proper laboratory quarters have been provided and no laboratory equipment, but an exceedingly minimum salary has been paid to a practicing physician in Rochester, who has provided the necessary equipment himself and done the work of the county laboratory, as well as could be expected with such half-way compromise. The personality and quality of the bacteriologist in this case has been of the highest class, and it is due to unusually well directed effort on his part that so creditable a showing has been made. "It is learned, however, that the services of this highly qualified and proven bacteriologist have now ceased and that a new man has taken over the work under similar conditions of private contract."

*Bulletin State Dept. of Health.*

Course of lectures in the out-patient hall of the N. Y. Skin and Cancer Hospital Wednesday afternoons, March 13, to April 17, at 4.15 o'clock. Diet and Hygiene in Diseases of the Skin, Dr. Bulkley.

April 24, Some Recent Methods of Treatment for Malignant Diseases, Dr. Bainbridge.

Each lecture will be preceded by a half hour Clinical Demonstration of various cases.

The lectures will be free to the Medical Profession, on the presentation of their professional cards.

---

In New York City, during 1911, 423 persons were killed and 2,004 injured by traffic. For 1910, the figures were: 376 and 930. Contrary to popular belief, automobiles do not kill as many persons as wagons—142 against 172. Nearly half—60—automobile homicides were attended with escape of the chauffeur. In this connection it may be noted that for several years, the number of persons killed in and by automobiles is approximately equal to the number killed by railroads and trolleys, for the whole country.

---

Ambrose Moore, who died Feb. 13, 1912, at his home in Mt. Carbon, Pa., aged 40, of kidney disease, weighed 525 pounds, and had a waist measure of 5 feet From Bridgeport, Conn., comes the report of a death ascribed to excessive adiposity. Within a couple of years, the patient had increased from "slight build" to nearly 400 pounds.

---

An Atlantic City man, 5 feet 6 inches tall, has just died, weighing 530 pounds. Ten years ago he weighed 143.

---

The best and largest library in the world, devoted to a single subject, is that of the Surgeon-General of the U. S. Army, which began with a small collection by Surgeon-General Lovell prior to 1836 but owed its existence on a large scale to Dr. John Billings in 1865.—(F. J. Haskin, *Buffalo Commercial*).

---

The Rochester Common Council, with the approval of the Public Health Association, is considering an ordinance against the public cup and towel.

